# COVID-19 vaccine and the risk of flares in inflammatory arthritis: a systematic literature review and meta-analysis

**DOI:** 10.3389/fimmu.2024.1503895

**Published:** 2024-11-01

**Authors:** Ariela Hoxha, Giovanni Striani, Marco Lovisotto, Paolo Simioni, Andrea Doria, Roberta Ramonda

**Affiliations:** ^1^ Thrombotic and Haemorrhagic Diseases Unit, Department of Medicine (DIMED), Internal Medicine Division, Padova University Hospital, Padova, Italy; ^2^ Rheumatology Unit, Department of Medicine (DIMED), Padova University Hospital, Padova, Italy

**Keywords:** inflammatory arthritis, COVID-19 vaccination, rheumatoid arthritis, spondyloarthritis, SARS-CoV-2 vaccine, AEFI

## Abstract

**Introduction:**

Coronavirus disease 2019 (COVID-19) vaccines aroused concerns about the risk of flares and adverse events in inflammatory arthritis (IA) since the vaccine clinical trials did not specifically investigate this subset of patients.

**Methods:**

A systematic literature review and meta-analysis to summarize the data on joint disease flare and adverse events following immunization (AEFI). Two researchers independently evaluated the literature on Pubmed, Scopus, and EMBASE databases from 22^nd^ March 2020 to 30^th^ September 2023. A random-effects model was used to pool odds ratios (OR) (with 95% CI) for the risk of joint disease flares and adverse events. Subgroup analyses were performed to evaluate the risk of disease flare between different IA and adverse events. Heterogeneity was assessed by I^2^ statistic.

**Results:**

A total of 9874 IA patients were included in the study: 6579 (66.6%) patients affected by RA and 3295 (33.4%) spondyloarthritis (SpA). The overall rate of flares was higher in RA vs. SpA (9.1% vs. 5.3%). However, the pooled estimated analysis showed no increased risk of joint disease flare following COVID-19 vaccination in patients affected by RA vs. SpA [OR 0.88, 95% CI: 0.77-1.00]. Furthermore, a subgroup analysis showed an increased risk of joint flares in psoriatic arthritis (PsA) patients vs. RA [OR 0.79, 95% CI: 0.68-0.93, p=0.004]. The pooled estimated analysis revealed no increased risk of AEFI in patients with RA vs. SpA [1.02, 95% CI: 0.63-1.65].

**Conclusions:**

Our meta-analysis summarized the current evidence on joint disease flares and COVID-19 vaccine-associated AEFI in IA patients. Pooled analysis showed an increased risk of disease flares in PsA vs. RA patients.

## Highlights

- No increased risk of joint disease flares associated with COVID-19 vaccines in patients affected by RA vs. SpA.
- Higher risk of joint disease flares in RA patients vs. PsA patients.
- No difference in the pooled estimated analysis of AEFI in any IA subsets.

## Introduction

The Coronavirus disease 2019 (COVID-19) pandemic and the following vaccination campaigns raised concerns about a possible increased risk of flares and adverse events following immunization (AEFI) in patients with inflammatory arthritis (IA), bearing in mind that COVID-19 vaccine clinical trials patients did not specifically investigate this subset of patients.

Over the past two decades, significant progress has been made in understanding the role of vaccines in preventing infectious diseases, particularly in individuals with IA who may be at higher risk of infectious events due to their underlying conditions, comorbidities, and immunosuppressive treatments (1–8). Although live vaccines are generally avoided in most patients with IA, live-attenuated vaccines — i.e. influenza, pneumococcal disease, and hepatitis B — are considered safe and effective for these patients (9). The current literature data focuses mainly on immunogenicity, showing no or little effect of immunosuppressive drugs (10–15). Some recently reviewed data from low-level evidence studies (16) did not show a higher rate of joint disease flares and AEFI. Data on COVID-19 vaccination in IA, mainly from retrospective studies, showed varying rates of disease flares, from 0 to 21.9% (17, 18). However, flares were often mild and easily manageable with non-steroid anti-inflammatory drugs (NSAIDs) or short courses of steroid therapy (17–27). In a recently published study investigating AEFI in a cohort of patients with IA, we found that 52 (15.7%) patients developed joint disease flares within one month of COVID-19 vaccination; 12 (23.1%) required a switch to another therapy, whereas the disease flare was successfully managed with short-course NSAIDs in 29 (55.8%) and with short-course corticosteroids in 11 (21.1%) (23). Patients who experienced disease flare had a higher disease activity than those without a disease flare (30.8% vs. 20.1%) (26).

Regarding AEFI in auto-immune inflammatory rheumatic disease, a *post hoc* analysis of two parallel randomized clinical trials (RCT) of adjuvanted recombinant herpes zoster vaccine showed similar rates of severe adverse events (AEs) and fatal severe AEs between herpes zoster vaccine and placebo (28). Few studies evaluated the prevalence of AEs following SARS-CoV-2 vaccination, but all concurred that the AEFI rate is comparable to that observed in healthy controls (26, 29–34). Nevertheless, we did find an even greater incidence of AEFI in our control group comprising healthy subjects (26). It bears noting that these AEFIs are mostly mild, short-lived, and self-limiting; reports of severe AEFI are rare and comparable to that observed in healthy controls (30–34).

Therefore, we conducted a systematic literature review and meta-analysis to summarize the latest data on joint disease flare and adverse events following COVID-19 vaccine to provide vital information to healthcare professionals caring for patients with IA.

## Materials and methods

### Data sources and search strategy

We performed a systematic literature review following the Preferred Reporting Items for Systematic Reviews and Meta-Analyses (PRISMA) guidelines as reported in **Supplementary Table 1** (35). Two researchers (HA and SG) independently evaluated the literature on Pubmed, Scopus, and EMBASE databases from the 22^nd^ of March 2020 to the 30^th^ of September 2023. The search strategy combined free text search exploded medical subject headings (MESH/EMTREE) terms and all synonyms of the following MESH: “anti-SARS-CoV-2 vaccine” OR “anti-SARS-CoV-2 vaccination” OR “COVID-19 vaccine” OR “COVID-19 vaccination” AND “inflammatory arthritis” OR “psoriatic arthritis” OR “rheumatoid arthritis” OR “axial spondyloarthritis” OR “ankylosing spondylitis”.

### Eligibility criteria

The inclusion criteria, determined before the data search, were: 1) RCT and observational cohort study evaluating the incidence of disease flare and/or AEFI following COVID-19 vaccination; 2) study cohort consists of patients with the diagnosis of rheumatoid arthritis (RA) and/or psoriatic arthritis (PsA) and/or ankylosing spondylitis (AS) who received vaccination. All studies that did not meet the criteria for study design, characteristics of the study population, or outcome were excluded.

### Selection process

**Figure 1:**
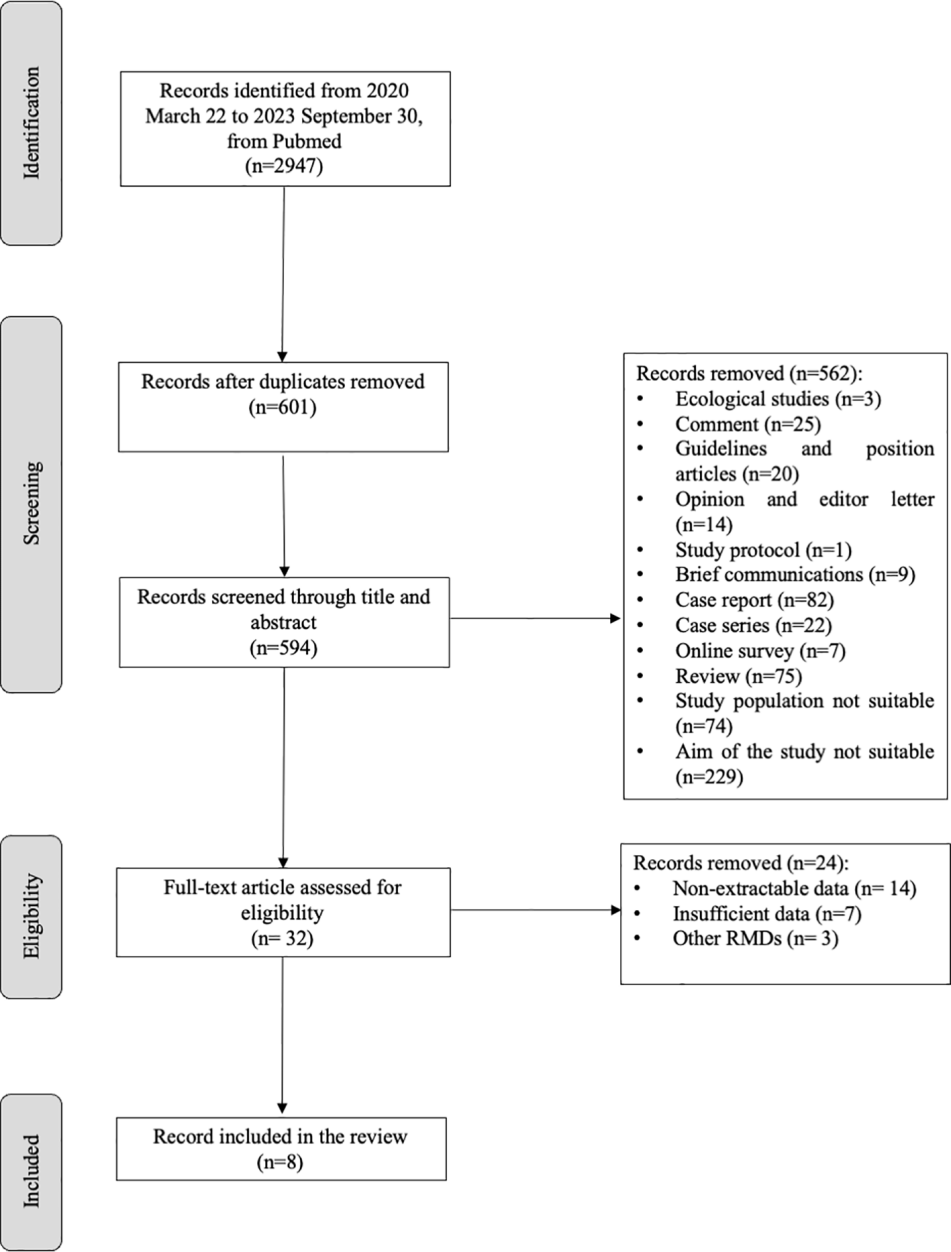
Flow diagram overview of study selection.

**Figure 1** shows the flow chart of the study selection. According to the PRISMA guidelines (35), 594 articles were screened through title and abstract after removing duplicate and non-English records. Five-hundred-sixty-two articles were removed for the not-eligible study design; 32 were screened through full text, and eight were selected for meta-analysis. **Table 1** illustrates a summary of the characteristics of the selected studies. Two researchers independently extract the data in an *ad hoc* electronic form. For each included study, year of publication, study design, sample size, rheumatic inflammatory arthritis, and type of vaccination were recorded, as well as the following clinical manifestations: disease flare, AEFI, fever, asthenia, and arthralgia. The definitions for disease flare, AEFI, fever, asthenia, and arthralgia are reported in **Supplementary Table 2**. Furthermore, a 2x2 table was elaborated for each study based on vaccination status and clinical manifestation.

**Table 1:** Characteristics of the studies included in this systematic review and meta-analysis.

Authors, year	Study design	Sample size IA, n	Type of IA, n (%)	Type of vaccine	Vaccinated IA patients°, n (%)	IA flare*, n (%)	AEFI*, n (%)
Striani, 2023 (35)	Retrospective	362	AS 110 (30.4), PsA 158 (43.6), RA 94 (26.0)	Comirnaty, Spikevax, Vaxzevria	331 (91.4)	52 (15.7)	102 (30.8)
Spinelli, 2022 (36)	Prospective	66	AS 9 (13.6), PsA 26 (39.4), RA 31 (47.0)	NR	66 (100)	3 (4.5)	NR
Geisen, 2021 (37)	Prospective	13	AS 3 (23.1), PsA 2 (15.4), RA 8 (61.5)	Comirnaty, Spikevax	13 (100)	0 (0.0)	NR
Zamani, 2023 (38)	Prospective	59	AS 21 (35.6), RA 38 (64.6)	BBIBP‐CorV	59 (100)	NR	27 (45.8)
Álvaro-Gracia, 2023 (29)	Prospective	1765	PsA 587 (33.3), RA 1178 (66.7)	Comirnaty, Spikevax, Vaxzevria, Janssen	1765 (100)	70 (4.0)	NR
Fong, 2023 (39)	Retrospective	2377	AS 399 (16.8), PsA 415 (17.5), RA 1563 (65.7)	Comirnaty, Spikevax	2377 (100)	520 (21.9)	NR
Rider, 2022 (40)	Retrospective	2296	AS 291 (12.7), PsA 304 (13.2), RA 1701 (74.1)	Comirnaty, Spikevax, Vaxzevria, other unspecified	2296 (100)	118 (5.1)	NR
Ma, 2022 (41)	Retrospective	2936	AS 484 (16.5), PsA 486 (16.5), RA 1966 (67.0)	Comirnaty, Spikevax	2936 (100)	639 (21.8)	NR

IA, inflammatory arthritis; AEFI, adverse events following immunization; AS, ankylosing spondylitis; PsA, psoriatic arthritis; RA, rheumatoid arthritis; NR, not reported.

°At least the first dose of the vaccination cycle.

*The respective definitions of flare and AEFI for each study are reported in **Supplementary Table 1**.

All discrepancies were resolved by consensus between the two principal investigators. A third reviewer (RR) made the final decision.

### Assessment of methodological quality and heterogeneity

The Newcastle-Ottawa Scale (36) evaluates selection, comparability, and outcome to assess the quality of the studies included in the review. Furthermore, the Agency for Healthcare Research and Quality (AHRQ) thresholds were used for the conversion.

### Statistical analysis

The meta-analysis used the Review Manager Review Manager (RevMan) [Computer program]. Version 5.4. The Cochrane Collaboration, 2020. The meta-analysis was performed after assessing the homogeneity of the study designs, populations, and outcomes. In the primary analysis, we evaluate the risk of flare following COVID-19 vaccination in RA and SpA patients. The risk of developing adverse events following COVID-19 vaccination in RA and SpA patients was also evaluated. We used the odds ratio (OR) as the measure of association in this meta-analysis. Pooled risk estimates were obtained using a random-effects model with restricted maximum likelihood (REML) estimations to calculate 95% prediction intervals. Heterogeneity between studies was calculated using the I2 (0–100%). According to the Cochrane Handbook, an I2 ≥ 75% or higher represents considerable heterogeneity; thus, pooled results were exclusively reported if I2 was below 75% (37). Publication bias was examined using a funnel plot and Egger’s regression test (38). Separate *a priori* subgroup analyses were planned to evaluate the risk of fever, arthralgia, and asthenia. Descriptive analysis was conducted using STATA version 17 software (StataCorp. 2021. Stata Statistical Software: Release 17. College Station, TX: StataCorp LLC). Data are shown as numbers (percentages).

## Results

### Literature flow chart and study characteristics

We retrieved 2947 articles using Pubmed, Scopus, and EMBASE databases from 2020 March 22 to 2023 September 30. After removing duplicates and not English papers (n=7), and screening the studies through title and abstract, among the 32 remaining studies, 24 were excluded for non-extractable data (n=14), insufficient data (n=7), and other RMDs (n=3). We included eight articles (17, 18, 20, 23, 26, 29, 39, 40) in the quantitative and qualitative analysis (**Figure 1**; **Table 1**). These were all cohort studies, four prospective (18, 20, 29, 39) and four retrospectives (17, 23, 26, 40).

### Patients’ characteristics

A total of 9874 IA patients were included in the study: 6579 (66.6%) patients with RA and 3295 (33.4%) with spondyloarthritis (SpA). Among the latter, 1317 (40.0%) had AS and 1978 (60.0%) had PsA. Nine-thousand-eighty-hundred-forty-three (99.7%) patients received a COVID-19 vaccine: 6567 (66.7%) with RA and 3276 (33.3%) with SpA, the latter of whom 1312 (40.0%) had AS and 1964 (60.0%) PsA. Only one study (35) also included unvaccinated patients.

### Assessment of quality score and publication bias

**Figure 2:**
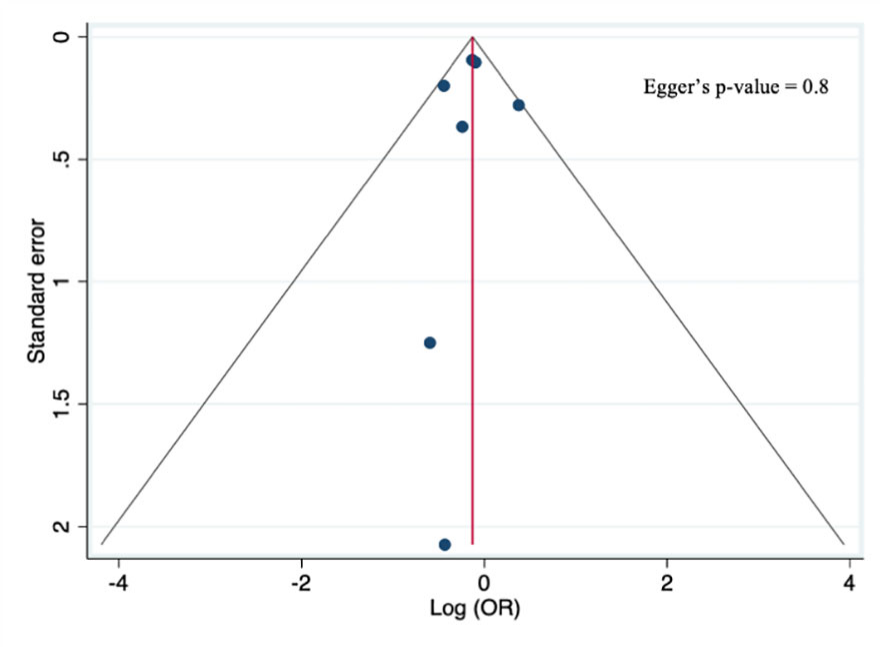
Funnel plot for the primary analysis.

The quality score assessment, as reported in **Supplementary Table 3**, was assessed using the Newcastle–Ottawa scale for cohort studies (36), which assigned a high-quality rating to five studies (18, 20, 26, 29, 40), a moderate quality to one (21), and a low-quality rating to two studies (17, 39). Furthermore, a visual examination of the funnel plots for the primary analysis showed no asymmetry, and Egger’s test (p=0.8) did not reveal any statistical evidence for publication bias, as shown in **Figure 2**.

### Risk of joint disease flares following COVID-19 vaccination in inflammatory arthritis

Seven studies evaluated the prevalence of joint disease flares following COVID-19 vaccination: 1413 (14.4%) vaccinated patients out of 9784 experienced a flare-up at the follow-up visit or self-reported flare within one month of vaccination, lasting for at least two days and requiring a change in treatment.

**Figure 3:**
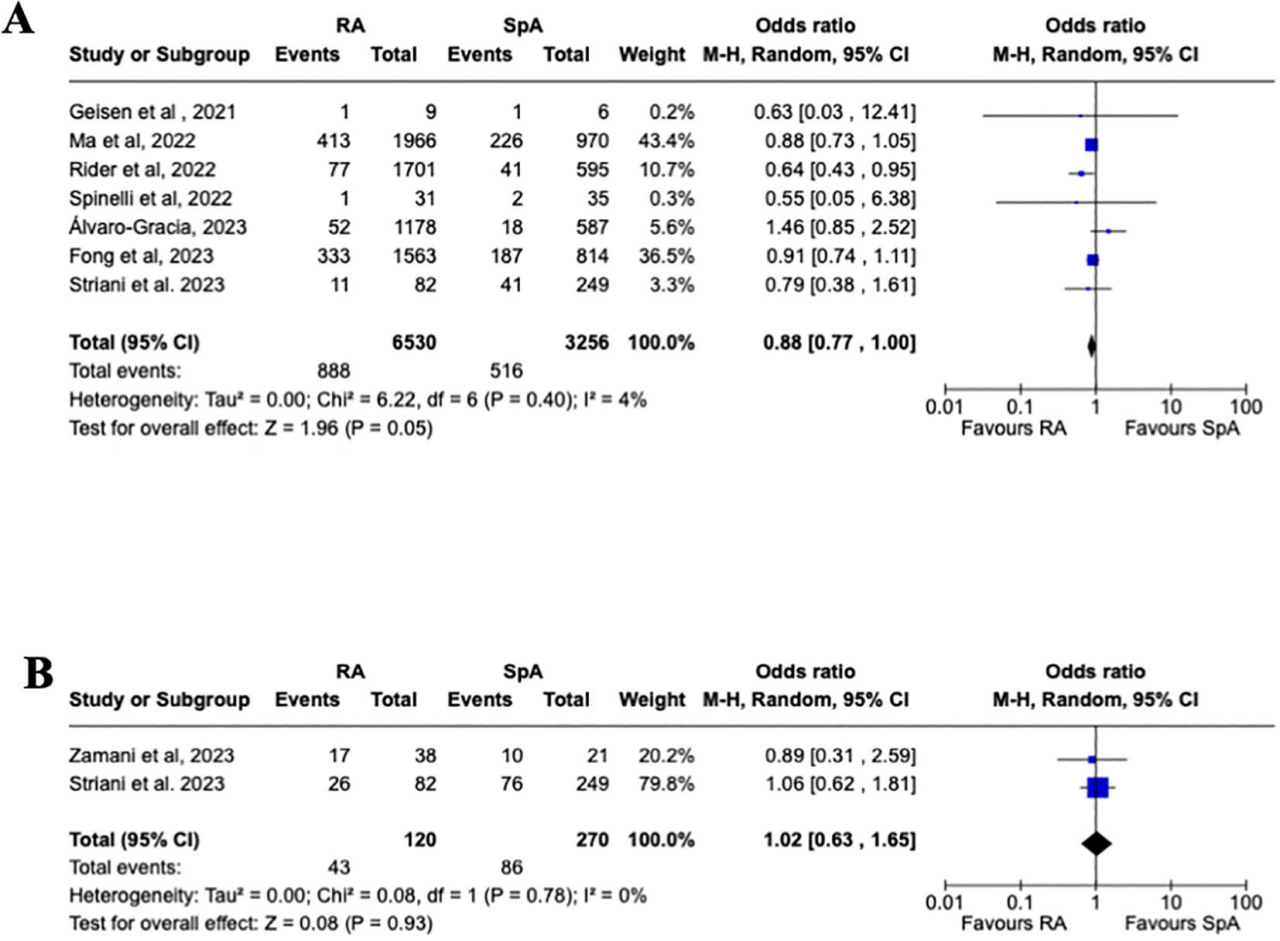
Primary analysis. **(A)** Forest plot for the risk of joint disease flares after receiving a COVID-19 vaccine in patients with rheumatoid arthritis and spondyloarthritis, **(B)** Forest plot for the risk of adverse events after receiving a COVID-19 vaccine in patients with rheumatoid arthritis and spondyloarthritis. Pooled ORs for flare **(A)** and adverse events **(B)** are reported, and the 95% confidence interval, number of pooled studies, number of observations, heterogeneity, and test of overall effect are presented.

**Figure 4:**
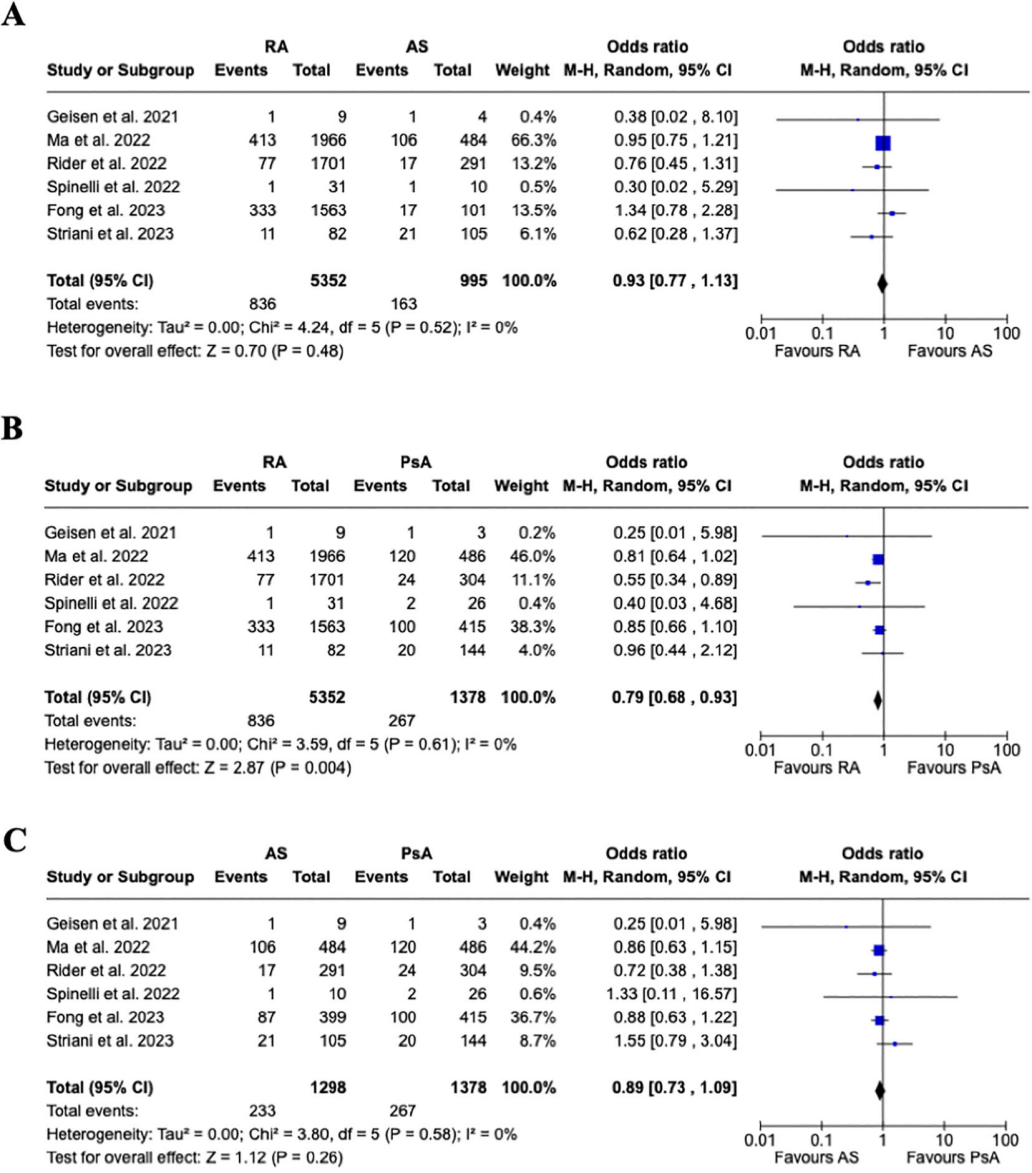
Subgroup analysis. **(A)** Forest plot for the risk of joint disease flare after receiving a COVID-19 vaccine in patients with rheumatoid arthritis vs. ankylosing spondylitis, **(B)** Forest plot for the risk of joint disease flare after receiving a COVID-19 vaccine in patients with rheumatoid arthritis vs. psoriatic arthritis, **(C)** Forest plot for the risk of joint disease flare after receiving a COVID-19 vaccine in patients with ankylosing spondylitis vs. psoriatic arthritis. Pooled ORs for flares are reported, and the 95% confidence interval, number of pooled studies, number of observations, heterogeneity, and test of overall effect are presented.

Even though the overall rate of flares was higher in RA vs. SpA (9.1% vs. 5.3%), the pooled estimated analysis (**Figure 3A**) shows no increased risk of joint disease flares following COVID-19 vaccination in patients affected by RA vs. SpA [OR 0.88, 95% CI: 0.77-1.00]. Furthermore, a subgroup analysis (**Figures 4A–C**) evaluating the risk of disease flare in the different IA conditions showed a reduced risk of joint flares in RA vs. PsA patients [OR 0.79, 95%CI: 0.68-0.93, p=0.004]. Nevertheless, no differences were found between RA vs. AS, and AS vs. PsA.

### Risk of COVID-19 vaccine-associated AEFI in inflammatory arthritis

Two studies evaluated the prevalence of AEFI following COVID-19 vaccination. Among 390 enrolled vaccinated patients, 129 (33.0%) patients experienced AEFI. Fifty-six (14.4%) developed fever, 44 (11.3%) arthralgia, and 65 (16.7%) asthenias. One death was reported in a patient affected by RA (26) following COVID-19, despite previously receiving two doses of the BNT162b2 (Pfizer-BioNTech) COVID-19 vaccine.

**Figure 5:**
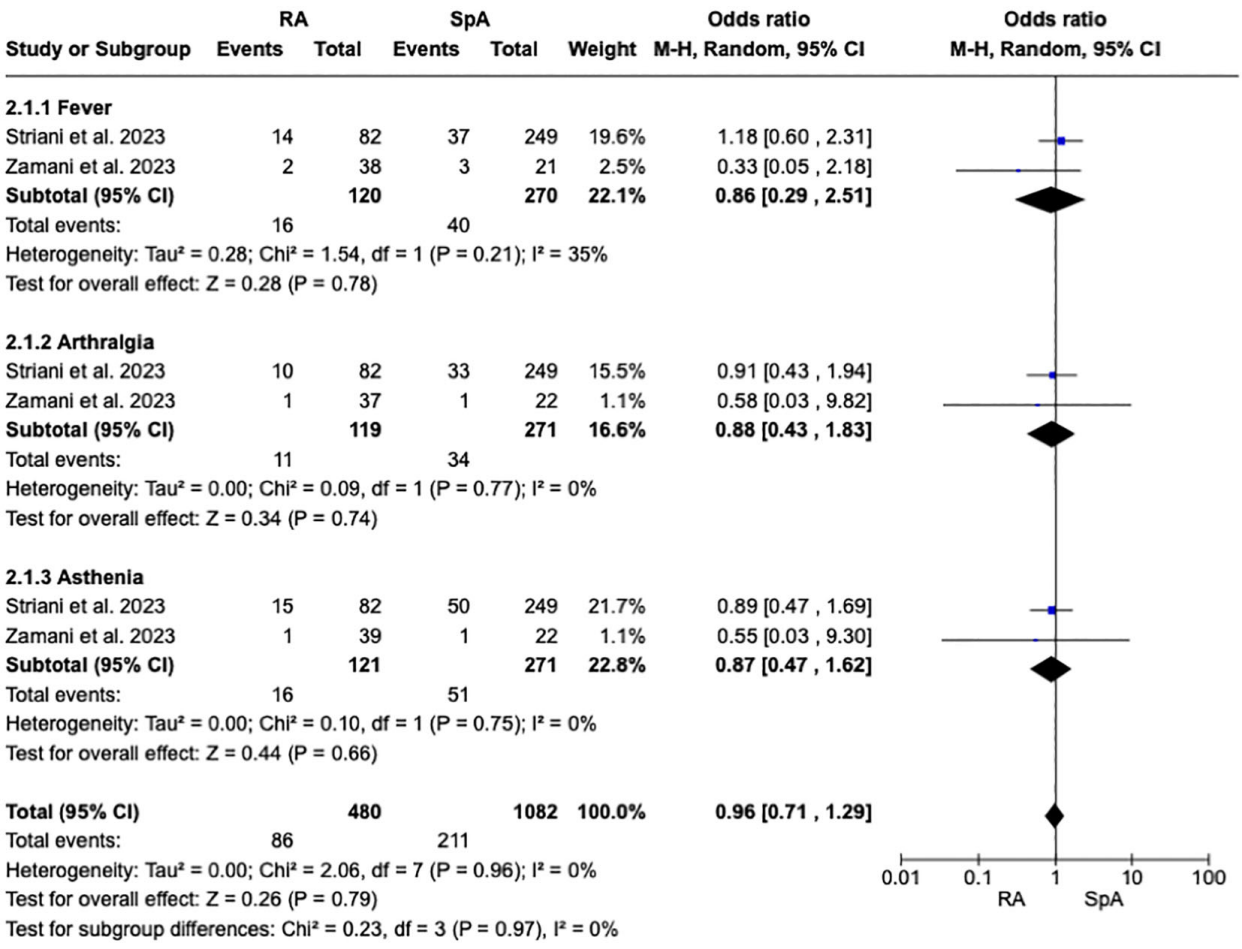
Forest plot for the subgroup analysis of adverse events, fever, arthralgia, and asthenia after receiving a COVID-19 vaccine in patients with rheumatoid arthritis and spondyloarthritis. Pooled ORs for adverse events are reported, and the 95% confidence interval, number of pooled studies, number of observations, heterogeneity, and test of overall effect are presented.

As reported in **Figure 3B**, a pooled estimated analysis did not reveal an increased risk of AEFI in patients affected by RA vs. SpA [1.02, 95% CI: 0.63-1.65]. Moreover, the subgroup analysis evaluating the different AEFI did not show an increased risk between the different IA, as reported in **Figure 5**.

## Discussion

This meta-analysis estimated the risk of joint disease flares and COVID-19 vaccine-associated AEFI in patients with different types of inflammatory arthritis. Overall, we found an estimated 14.4% rate of joint disease flares within one month of vaccination, 9.1% in patients affected by RA and 5.3% in those with SpA. There was no increased risk of joint disease flares associated with COVID-19 vaccines in patients affected by RA vs. SpA. This aligns with previous findings of 5-11% rate of disease flares following influenza vaccination in RA, depending on methotrexate (MTX) discontinuation (41), albeit with similar disease activity in both groups. Furthermore, an RCT investigated the timing of MTX discontinuation in RA patients and found that the rates of flares in continued MTX, discontinued MTX for four weeks before vaccination, discontinued MTX for two weeks before and two weeks after vaccination, and discontinued MTX for four weeks after vaccination were 24.1%, 21.2%, 34.1%, and 38.8%, respectively (42),. No significant differences in disease activity were observed between all 4 groups. There is scarce data on COVID-19 vaccine-associated disease flare in SpA, which mostly focuses on PsA. In a cohort study comprising 63 PsA patients treated with tumor necrosis alpha inhibitors, there were no significant changes in the tender and swollen joint counts, dactylitis, psoriasis area and severity index (PASI) score, patient-reported outcomes and evaluation by the treating physician, and erythrocyte sedimentation rate (ESR) (43).

Interestingly, we found that subgroup analysis revealed a lower risk of flares in RA patients vs. PsA patients, whereas there was no increased risk of flares in AS and PsA patients. The current literature lacks data comparing the impact of vaccines on disease activity in RA, PsA, and AS. A case-control study of 25 vaccinated vs. 25 non-vaccinated PSA patients showed a significantly increased tender joint count (TJC) and ESR at T1 (one month from vaccination), and TJC at T3 (three months from vaccination) (44). Thus, we speculate that patients with RA may exhibit a lower flare rate as they are usually treated with a higher rate of combination treatment (MTX+ bDMARDs), than PsA.

Overall, we registered an estimated rate of 33% of AEFI in vaccinated patients. There was no difference in the pooled estimated analysis of AEFI in any IA subsets. However, a meta-analysis comprising 13 studies concluded that local, mild AEs occurred significantly more frequently (RR = 1.77, 95% CI 1.02 to 3.08, *p* = 0.04) in RA patients than in healthy subjects (45). To note, that the AEFIs recorded were mild and self-limiting and did not require hospitalization. Recently, an interplay between SARS-CoV-2 and the host has been hypothesized, which may induce immune-mediated reactions, probably induced by the anti-spike antibodies, leading to immune-mediated diseases (46).

We would be remiss not to mention some of the limitations of our study. Firstly, the retrospective design and the relatively small number of studies included in this meta-analysis. Secondly, the sample size of studies may also have influenced the fidelity of the results concerning AEFI. Furthermore, we were not able to perform pooled analyses on ongoing treatments due to missing data. However, among the strengths of our study, the studies included in the meta-analysis did not reveal any statistical evidence of publication bias.

In conclusion, a pooled analysis showed a reduced risk of disease flares in RA vs. PsA patients, leading to speculation regarding the impact of treatment particularly MTX. Prospective-powered studies are needed to clarify the role of MTX on flares and immunogenicity in IA, thus guiding healthcare professionals toward optimal patient care and vaccination strategies in patients affected by IA.

## Data Availability

The raw data supporting the conclusions of this article will be made available by the authors, without undue reservation.
